# Urbanization as a risk factor for aortic stiffness in a cohort in India

**DOI:** 10.1371/journal.pone.0201036

**Published:** 2018-08-01

**Authors:** Laura Corlin, Kevin J. Lane, Jahnavi Sunderarajan, Kenneth K. H. Chui, Harivanza Vijayakumar, Lawrence Krakoff, Anbarasi Chandrasekaran, Sadagopan Thanikachalam, Doug Brugge, Mohan Thanikachalam

**Affiliations:** 1 Department of Civil and Environmental Engineering, Tufts University School of Engineering, Medford, Massachusetts, United States of America; 2 Department of Environmental Health, Boston University School of Public Health, Boston, Massachusetts, United States of America; 3 Agada Hospital, Chennai, India; 4 Department of Public Health and Community Medicine, Tufts University School of Medicine, Boston, Massachusetts, United States of America; 5 PURSE-HIS Study Center, Sri Ramachandra University, Chennai, India; 6 Mount Sinai Icahn School of Medicine at Mount Sinai, New York, New York, United States of America; 7 Tisch College of Civic Life, Tufts University, Medford, Massachusetts, United States of America; Hospital Universitari i Politecnic La Fe, SPAIN

## Abstract

Urbanization is associated with higher prevalence of cardiovascular disease worldwide. Aortic stiffness, as measured by carotid-femoral pulse wave velocity is a validated predictor of cardiovascular disease. Our objective was to determine the association between urbanization and carotid-femoral pulse wave velocity. The analysis included 6166 participants enrolled in an ongoing population-based study (mean age 42 years; 58% female) who live in an 80 × 80 km region of southern India. Multiple measures of urbanization were used and compared: 1) census designations, 2) satellite derived land cover (crops, grass, shrubs or trees as rural; built-up areas as urban), and 3) distance categories based on proximity to an urban center. The association between urbanization and carotid-femoral pulse wave velocity was tested in sex-stratified linear regression models. People residing in urban areas had significantly (p < 0.05) elevated mean carotid-femoral pulse wave velocity compared to non-urban populations after adjustment for other risk factors. There was also an inverse association between distance from the urban center and mean carotid-femoral pulse wave velocity: each 10 km increase in distance was associated with a decrease in mean carotid-femoral pulse wave velocity of 0.07 m/s (95% CI: -0.09, -0.06 m/s). The association was stronger among older participants, among smokers, and among those with other cardiovascular risk factors. Further research is needed to determine which components in the urban environment are associated with higher carotid-femoral pulse wave velocity.

## Introduction

Over half of the world’s population resides in urban areas and this proportion may increase to 66% by 2050 [1]. Up to 90% of the projected increase is due to accelerated urbanization in Africa and Asia [1]. Currently, the world’s second largest urban population resides in India with approximately 410 million people and this number is projected to double by 2050 [1]. With increasing urbanization, there are concerns about an increasing prevalence of cardiovascular disease (CVD), a leading cause of death worldwide [2]. Urbanization is one of the major upstream socio-environmental factors associated with the rise in CVD and increases in cardiometabolic risk markers in rapidly developing countries [3–6]. The urbanization process itself, whereby municipalities undergo growth in population density and complexity [4], leads to shifts in diet, physical activity, and psychosocial demands resulting in an increase in CVD risk factors [7–9]. In addition, urbanization may increase individuals’ exposure to air pollution and other environmental risk factors [10].

Attempts to reduce the morbidity and mortality of CVD in recent decades have focused increasingly on intermediate endpoints, such as aortic stiffness [11]. The “gold standard” assessment of aortic stiffness is the carotid-femoral pulse wave velocity (cfPWV), a commonly used measure of the velocity of the pulse wave moving from the heart to the carotid and the femoral artery [12]. Apart from the dominant effect of aging [13], other cardiovascular risk factors such as obesity [14,15], smoking [16,17], lack of physical activity [18,19], hypercholesterolaemia [20], and type 2 diabetes mellitus [21] are associated with increased cfPWV. High cfPWV can lead to increases in systolic blood pressure (SBP) and incident hypertension (HTN) [22,23]. High cfPWV is also associated with target organ damage including left ventricular hypertrophy [24], renal dysfunction [25], and increased white matter hyperintensity volume [26]. Furthermore, cfPWV is an independent predictor of all-cause and cardiovascular mortality, fatal and non-fatal coronary events, and strokes [11,27,28]. Several studies indicate that considering cfPWV in addition to standard CVD risk factors improves CVD risk prediction in the general population [29–31].

There is an increasing body of research investigating the association between CVD and urbanization, defined as land predominantly covered by man-made structures (built environment) or areas with intensive use [32]; however, there is a paucity of information on the association between urbanization and aortic stiffness. In particular, while elements within the urban environment, such as obesity, psychosocial stress, and other cardiovascular risk factors are known to be associated with both urbanization and with cfPWV, the association between built environment and cfPWV is not known. In the current study we examined the association between cfPWV and urbanization in a region of southern India. Chennai, the fourth largest metropolitan city in India (population >9 million; 2013 gross domestic product per capita of ~$2000; primary industries include software, hardware and electronic manufacturing, automotive, tourism, and entertainment) [33,34], served as the primary location from which the urban study population was recruited. The semi-urban and rural areas were near Chennai in the Thiruvallur and Kanchipuram districts, respectively. This is an important population to study given a hypertension prevalence of >40% in certain urban areas of India [35,36]. We defined urbanization using 1) population density in the India Census, 2) distance from the Chennai urban center, and 3) land cover type. We also examined whether sex, obesity, and other cardiovascular risk factors modified the association between urbanization and cfPWV. We used health data from the Population Study of Urban, Rural, and Semi-urban Regions for the Detection of Endovascular Disease and Prevalence of Risk Factors and Holistic Intervention Study (PURSE-HIS) [37].

## Methods

### Study population

The PURSE-HIS was designed and implemented to understand the prevalence and progression of subclinical and overt endovascular disease and its risk factors in urban, semi-urban, and rural communities in an 80 km x 80 km region of southern India. The detailed methodology of PURSE-HIS is published elsewhere [37]. Briefly, 8080 participants over 20 years of age were recruited from urban (n = 2221), semi-urban (n = 2821), and rural (n = 3038) areas in the state of Tamil Nadu, India through a two stage cluster sampling method to ensure adequate spatial variability amongst administrative divisions. In the first stage, urban administrative units, village-level administrative units, and rural administrative units were chosen. In the second stage, simple random sampling was used to choose streets (urban areas), wards (semi-urban areas), or villages (rural areas). The sample size was determined based on an assumption of 20% nonresponse, a design effect of 2, and coronary artery disease prevalence of 10% in urban areas and 7% in rural areas. We set the power at 80% and alpha at 5%.

In the current analysis, since both semi-urban and urban populations were similar in their cardiovascular risk and disease profile, they were combined and identified as semi-urban/urban. Participants could not have carcinomas, severe psychiatric illnesses, stage IV cardiac failure, or human immunodeficiency virus infection. We further excluded participants with a previous history of hypertension (HTN; n = 1115) or diabetes mellitus (DM; n = 921, of whom 355 also had a history of hypertension). Furthermore, 141 participants were excluded due to missing cfPWV values and 92 participants were excluded from the main analysis due to extreme cfPWV values (≥3 standard deviations away from the mean). A sensitivity analysis included these 92 participants with extreme cfPWV values.

The study was approved by the Institutional Ethics Committee (IEC-06/53/47) at Sri Ramachandra University (Chennai, India), by the Institutional Review Board at Tufts University (Boston, United States), and was registered with Clinical Trials Registry, India (CTRI/2011/04/001677). Participants gave written informed consent.

### Questionnaire and clinical data collection

An interviewer-administered questionnaire was used to collect data on CVD and its risk factors. After a general clinical examination, blood pressure (BP) of participants was measured by a trained physician in the dominant arm using a validated automated oscillometric BP device Omron Sem-1 (Omron Healthcare, Singapore) with an appropriate cuff size. Participants were seated with their arms at the heart level. Three readings were taken, each a minute apart, and the mean was used in the analysis. Participants with a measured SBP≥140 and/or diastolic BP (DBP) ≥90 were considered newly identified hypertensives. Physical activity was measured by a physiotherapist using the Global Physical Activity Questionnaire [38]. A sedentary score was calculated using this physical activity scale. A clinical psychologist assessed the level of stress and anxiety using the Presumptive Stressful Life Event Scale [39] and Hamilton Anxiety Rating Scale [40], respectively. A socioeconomic status (SES) score was computed based on the Kuppuswamy classification [41] taking into consideration education, occupation, and income status. Fasting blood specimens were collected and assayed for standard clinical lipid parameters [37]. Non-diabetics were given an oral glucose tolerance test [37]. Smoking status was recorded as current smoker or non-smoker.

### Carotid-femoral pulse wave velocity (cfPWV) assessment

Detailed methodology has been published previously [37]. By recording electrocardiography-gated carotid and femoral artery waveforms sequentially, the cfPWV was measured (SphygmoCor, AtCor Medical,West Ryde, New South Wales, Australia)[42]. The path length used to determine the cfPWV was measured with a tape measure as the surface distance between the suprasternal notch and femoral site. All measurements were made in duplicate by trained investigators.

### Geo-location and spatial variable creation

**Fig 1:**
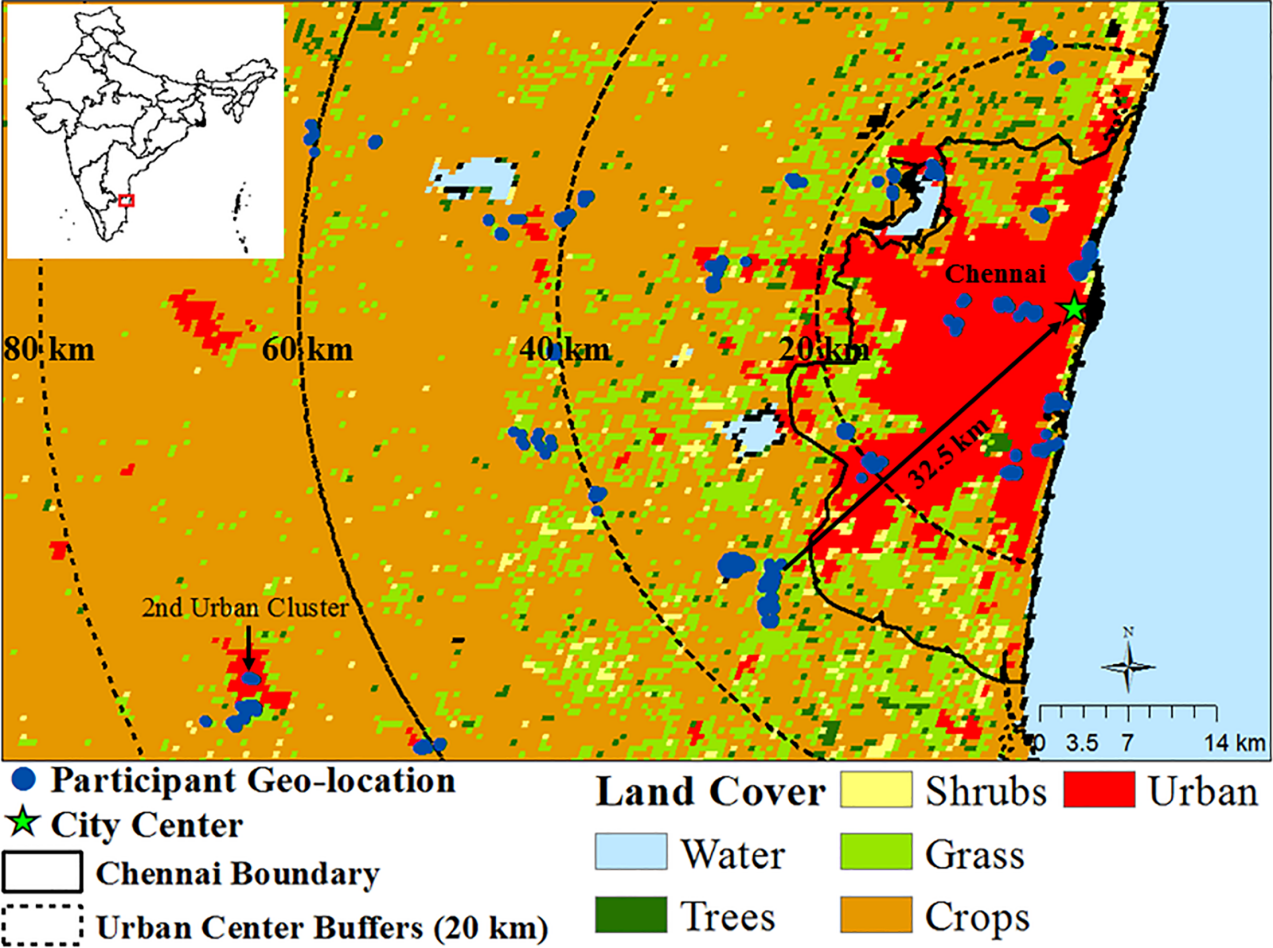
PURSE-HIS participants, distance categories, and land cover type. Participant locations are shown as blue dots, the Chennai city center is shown as a green star, the Chennai boundary is shown with a solid black line, and distance from the Chennai city center is noted with dashed lines at 20 km, 40 km, 60 km, and 80 km from the city center.

The urban center of the study region was defined as the flag post on the ramparts of the Fort Saint George historic landmark in Chennai, in accordance with historical and local custom (Fig 1). Residential addresses of study participants were geo-located to calculate distance (in kilometers) from the urban center for each participant using the Near Tool in ESRI ArcGIS v10.1 (Fig 1). Distance from Chennai urban center was calculated for each study participant and grouped into 0–20 km, 21–40 km, 41–60 km, and 61–80 km distance intervals for analysis.

### MODIS satellite-based land cover

The land cover data (MCD12Q1, NASA) were obtained through the online Data Pool at the NASA Land Processes Distributed Active Archive Center. The annual average values were derived from Terra and Aqua-MODIS land cover data products. The data presented are from 2010 and have 500 × 500 m resolution. The Plant Functional Scheme was used for classification of land cover type (Fig 1) [43]. Land cover data identified a second urban cluster (Fig 1) approximately 65 km southwest of the Chennai urban center (n = 305). Land cover groups were used to classify participants for comparison of mean cfPWV.

### Statistical analyses

Descriptive statistics, including means and standard deviations for continuous variables, were computed. T-tests and analysis of variance (ANOVA) were used to assess associations between relevant demographic characteristics, health indicators, and hemodynamic measures by sex and land cover designation. Cardiovascular risk factors were assessed for bivariate associations with cfPWV. Since there were significant sex differences in the prevalence of CVD and risk factors, sex-stratified multivariate models were constructed to assess the relationship between continuous distance to the urban center of Chennai and cfPWV. Models controlled for cardiovascular risk factors significantly associated with cfPWV including age (as a continuous variable), BMI, smoking (males only), heart rate, SES score, sedentary score, stress score, and anxiety score. Multivariable regression models for female participants did not control for smoker status because only two percent of women reported smoking.

To further assess the association between distance to the urban center and cfPWV, unadjusted models were stratified by clinical variables known to be major predictors of cfPWV including age group, smoking status, overweight/obesity status (BMI ≥ 25 kg/m^2^), newly diagnosed DM, and abnormal low density lipoprotein (LDL) [44,45]. One sensitivity analysis was completed adding in the 92 participants with extreme cfPWV values and a second sensitivity analysis was completed excluding the 183 participants who were genetic relatives of other participants. We also ran a sensitivity analysis to examine mean cfPWV differences between urban and non-urban land cover designated participants residing in the 60–80 km distance group as urban because of the presence of the second urban area. All models were checked for influential cases and collinearity. The normality and homoscedasticity of the standardized residual errors were assessed. All statistical analyses were done with SPSS version 18 or Stata v15.1. Associations with p < 0.05 were considered statistically significant.

## Results

Demographic characteristics of the 6166 participants are presented in Table 1. The mean age was 42 years (s = 9.9 years) and 58% of participants were female. There were small, but statistically significant differences between urban/semi-urban and rural populations (defined by census designation) in smoker status, SES score, anxiety score, stress score, sedentary scores, mean BMI, and mean LDL. The significantly higher prevalence of newly diagnosed DM in the urban/semi-urban population was present in both sexes. Overall, men had a higher prevalence of newly diagnosed DM than women. In addition, men were significantly more likely to be smokers (Table 1).

**Table 1:** Demographic and health characteristics of PURSE-HIS cohort stratified by sex and community type as defined by census designation (n = 6166).

Demographic	Female		Male	
	Urban/Semi-Urban	Rural	Urban/Semi-Urban	Rural
	% or mean (s)	% or mean (s)	% or mean (s)	% or mean (s)
	n = 1096	n = 2489	n = 784	n = 1797
Age				
20–41	62.0%	59.4%	46.4%	41.1%
42–75	38.0%	40.6%	53.6%	58.9%
Smokers^a^^,^^b^^,^^c^^,^^d^	3.6%	1.4%	27.0%	32.9%
SES Score^a^^,^^b^^,^^c^^,^^d^^,^	12.9 (4.1)	12.2 (4.3)	14.2 (4.7)	13.7 (4.7)
Anxiety Score^a^^,^^c^^,^^d^^,^	8.1 (6.9)	6.8 (6.1)	6.0 (5.3)	5.8 (5.7)
Stress Score ^a^^,^^b^^,^^c^^,^	5.0 (3.0)	4.3 (3.1)	5.3 (2.7)	4.7 (3.0)
Sedentary Score^a^^,^^b^^,e^	4.9 (1.8)	4.7 (1.9)	5.0 (2.1)	4.6 (2.0)
New DM^a^^,^^b^	10.6%	7.8%	12.5%	9.2%
BMI (kg/m^2^)^a^^,^^b^^,^^c^^,^^d^	25.8 (4.7)	24.9 (4.8)	23.9 (4.2)	23.1 (4.1)
LDL(mg/dL)^a^^,^^b^	116.6 (32.7)	112.9 (31.0)	117.7 (31.1)	112.6 (31.5)
Heart rate (beats/minute)^c^^,^^d^	77.4 (13.8)	77.1 (12.7)	71.4 (13.5)	70.9 (13.3)
New HTN^a^^,^^b^^,^^c^^,^^d^	14.7%	9.1%	27.6%	16.2%
SBP (mmHg)^a^^,^^b^^,^^c^^,^^d^	120.6 (16.4)	113.8 (15.3)	127.5 (17.5)	118.6 (15.9)
DBP (mmHg)^a^^,^^b^^,^^c^^,^^d^	75.7 (10.5)	72.6 (9.6)	79.4 (11.1)	75.8 (10.3)
PP (mmHg)^a^^,^^b^^,^^c^^,^^d^	44.9 (12.7)	41.2 (10.6)	48.1 (12.0)	42.9 (11.1)

^a^ Indicates significant difference between urban and rural in females (p < 0.05).

^b^ Indicates significant difference between urban and rural in males (p < 0.05).

^c^ Indicates significant difference between rural males and rural females (p < 0.05).

^d^ Indicates significant difference between urban males and urban females (p < 0.05).

Abbreviations and scales: SES score = socioeconomic status (min = 3, max = 29), anxiety score (min = 0, max = 41), stress score (min = 0, max = 25), sedentary score (min = 0.16, max = 10), DM = diabetes mellitus, BMI = body mass index, LDL = low density lipoprotein, HTN = hypertension, SBP = systolic blood pressure, DBP = diastolic blood pressure, and PP = pulse pressure.

With the exception of mean heart rate, which was significantly higher in females, peripheral hemodynamic measures were all significantly higher in males than in females (Table 1). Regardless of sex, mean SBP, DBP, pulse pressure (PP), LDL cholesterol, and prevalence of newly diagnosed HTN were all significantly greater among participants living in urban/semi-urban areas compared to participants living in the rural areas.

**Fig 2:**
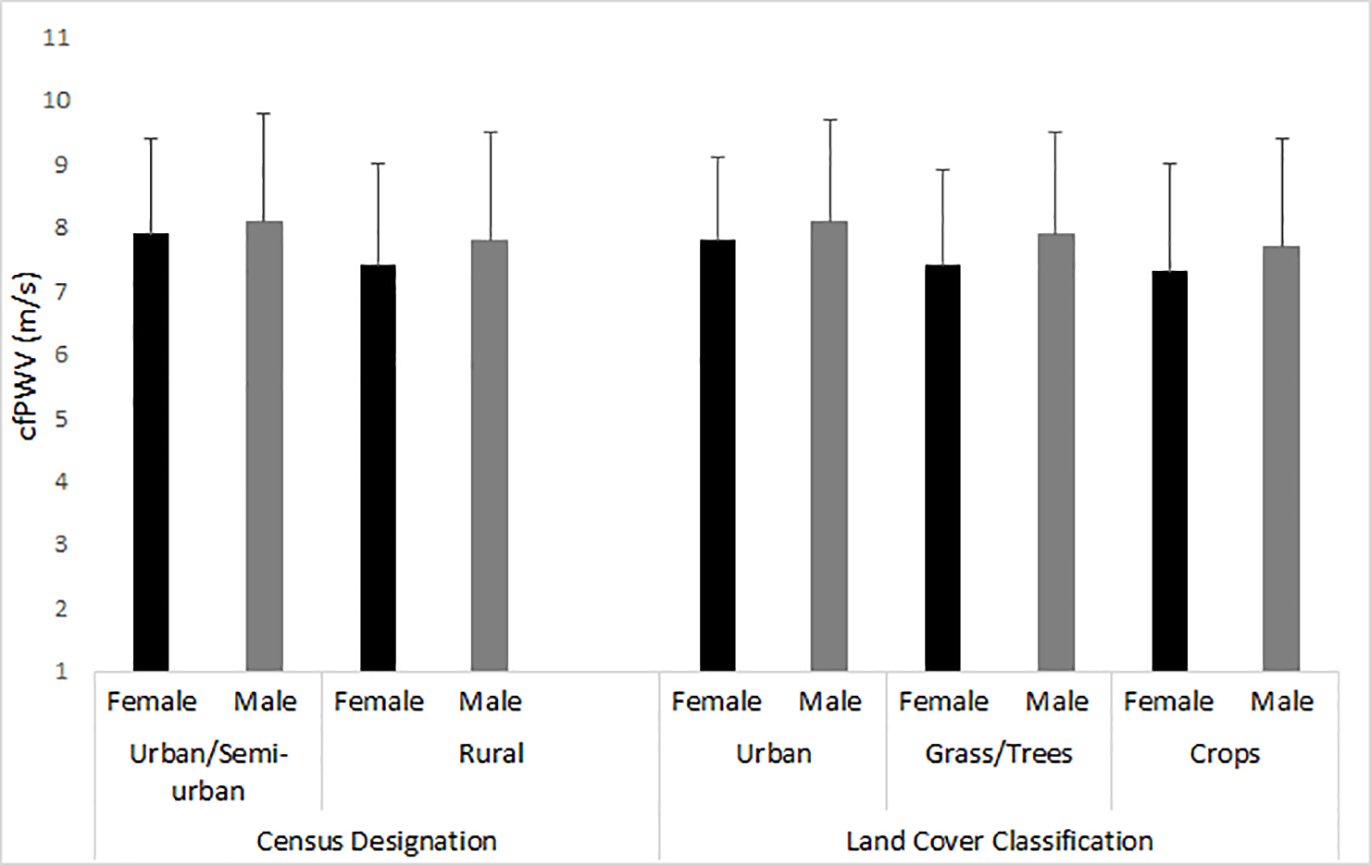
Census designation and land cover classification for mean carotid-femoral pulse wave velocity (cfPWV) by sex. Bars represent the standard deviation. For census designation, mean cfPWV is shown for urban/semi-urban and rural areas. For land cover classification, mean cfPWV is shown for urban areas, areas with grass/trees, and areas with crops. For all census and land cover comparisons, mean cfPWV is significantly (p < 0.05) higher in men than women. Mean cfPWV is significantly higher for men and women in urban areas than in rural areas as designated by the census or in areas with crops as designated by land cover. Mean cfPWV is also significantly higher for men in areas with grass/trees than in areas with crops.

Based on census data designations, mean cfPWV was significantly (p < 0.05) higher in the urban/semi-urban populations, compared to rural population (Fig 2). The mean cfPWV in urban/semi-urban women (7.6 m/s, s = 1.5) was significantly higher than the mean cfPWV in rural women (7.4 m/s, s = 1.6). Similarly, mean cfPWV was significantly higher in urban/semi-urban men (8.1 m/s, s = 1.7) than in rural men (7.8 m/s, s = 1.7). Based on the MODIS derived land cover classifications, in both sexes, participants residing in urban land cover had significantly elevated mean cfPWV compared to the mean cfPWV of participants residing in the non-urban land cover (crops; Fig 2).

**Fig 3:**
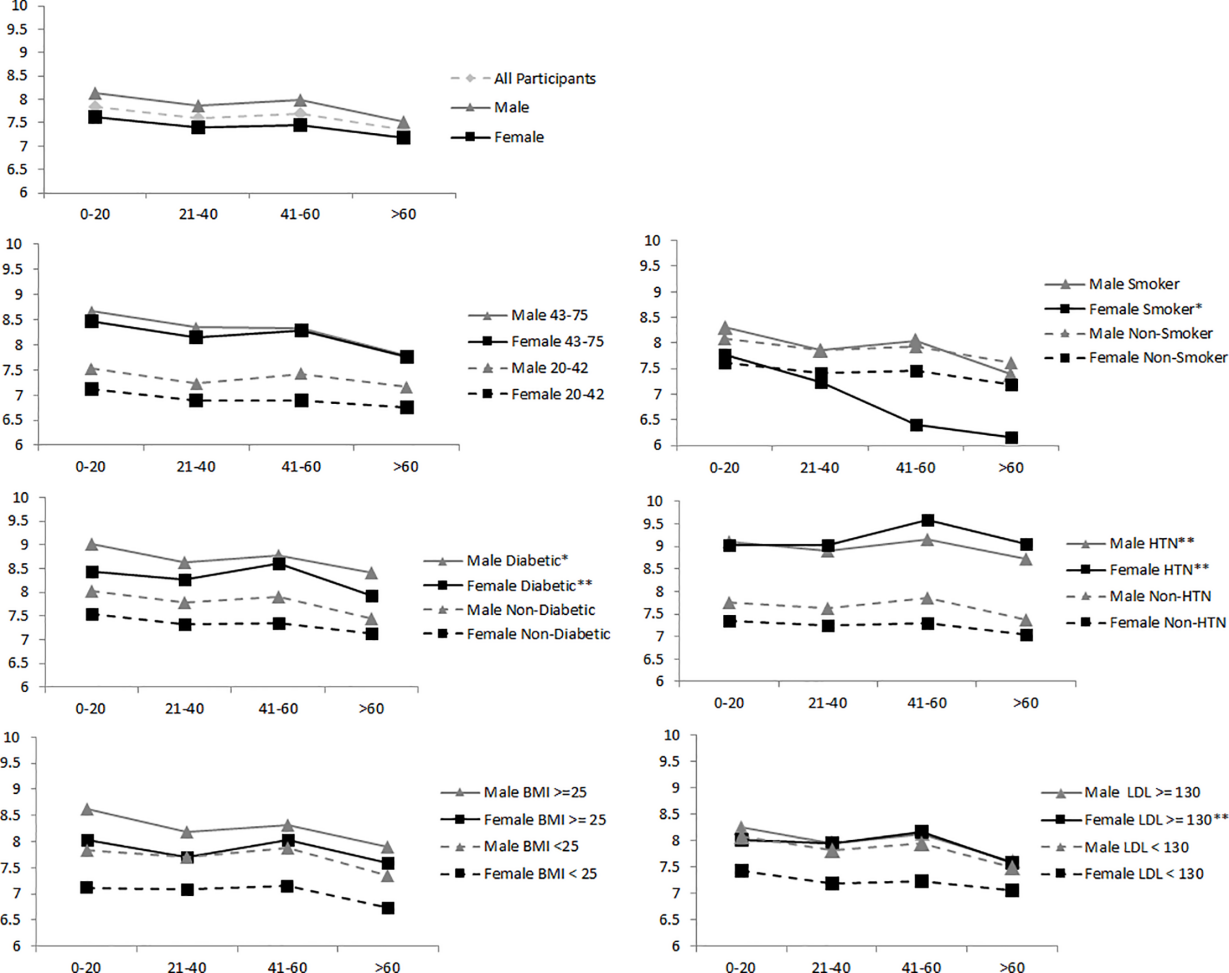
Mean unadjusted carotid-femoral pulse wave velocity (m/s) by 20 km distance intervals stratified by cardiovascular disease risk factors and sex. Lines with lighter symbols and triangle markers represent males and lines with darker symbols and square markers represent females. Solid lines represent groups that have higher levels of cardiovascular disease risk factors (e.g., older, smokers, or higher body mass index) while dashed lines represent groups that have lower levels of cardiovascular disease risk factors. HTN = hypertension; BMI = body mass index; LDL = low density lipoprotein. All tests for trends have p < 0.001 except those indicated by * (p < 0.05 but ≥ 0.001) or ** (p > 0.05).

In the overall population, the mean cfPWV was higher among participants residing closest to the Chennai city center than among participants residing 21–40 km, 41–60 km, or 61–80 km from the city center (Fig 3). The 61–80 km distance group had a significantly lower mean cfPWV than all other distance groups. These overall trends persisted in unadjusted analyses when stratified by age group, smoking, newly diagnosed diabetes, and overweight/obesity status. Within each distance group, the mean cfPWV was consistently higher for participants that were older (age greater than the mean of 42 years), diabetic, hypertensive, or overweight/obese in both males and females (Fig 3).

Using land cover type, we found that among participants residing in a rapidly expanding second urban cluster (n = 305; Fig 1), males had a significantly higher mean cfPWV (8.0 m/s, s = 1.8) compared to the non-urban male participants within the same distance group (7.5 m/s, s = 2.1). Among the female participants, those residing in the second urban cluster also had higher mean cfPWV (7.4 m/s, s = 1.4) than those not in the urban cluster but in the same distance group (7.1 m/s, s = 1.7), although this trend was not significant.

In sex-stratified multivariate analysis, distance from the urban center was a significant predictor of cfPWV. Each 10 km increase in distance was significantly associated with a 0.07 m/s decrease in mean cfPWV (Table 2). Among males, the association with distance from urban center was stronger in participants who were older and among smokers (Table 2). Among females, the association with distance was stronger in older participants. There were not differences in the effect estimates for men and women with newly diagnosed diabetes; however, the sample size was larger for women and the more precise confidence interval excluded zero. Controlling for mean arterial pressure weakened the effect estimates among diabetic women (p = 0.886), women with a BMI ≥ 25 (p = 0.976), and individuals with a LDL ≥ 130 (p = 0.283 for women and p = 0.056 for men). Controlling for age, glucose, BMI, LDL, smoking, sex and mean arterial pressure, each additional kilometer away from the urban center was associated with a 0.006 decrease in mean cfPWV (p < 0.001). When we used other measures for urbanization (census data or land use data) in models with all participants, we found that the mean cfPWV was higher for participants residing in urban areas compared to participants residing in other areas (p < 0.001 for all comparisons).

**Table 2:** Association between distance in kilometers from urban center and carotid-femoral pulse wave velocity (m/s).

	Female^a^		Male^b^	
	(β, 95% CI)	Adjusted-R^2,^ p	(β, 95% CI)	Adjusted-R^2,^ p
All participants	-0.007 (-0.009, -0.005)	0.290, <0.001	-0.007 (-0.010, -0.004)	0.213, <0.001
Age (years)				
20–41	-0.006 (-0.008, -0.004)	0.170, <0.001	-0.004 (-0.007, -0.001)	0.109, 0.022
42–75	-0.009 (-0.012, -0.005)	0.171, <0.001	-0.009 (-0.013, -0.005)	0.164, <0.001
New DM				
No	-0.007 (-0.009, -0.005)	0.282, <0.001	-0.007 (-0.009, -0.004)	0.209, <0.001
Yes	-0.008 (-0.015, -0.001)	0.201, 0.036	-0.008 (-0.018, 0.002)	0.106, 0.103
New HTN				
No	-0.005 (-0.007, -0.004)	0.244, <0.001	-0.003 (-0.006, -0.001)	0.176, 0.017
Yes	0.000 (-0.007, 0.007)	0.212, 0.958	-0.009 (-0.015, -0.002)	0.212, 0.013
BMI ≥ 25				
No	-0.007 (-0.010, -0.004)	0.291, <0.001	-0.006 (-0.009, -0.002)	0.199, 0.001
Yes	-0.006 (-0.009, -0.003)	0.271, <0.001	-0.008 (-0.013, -0.004)	0.215, <0.001
LDL ≥ 130				
No	-0.007 (-0.009, -0.004)	0.268, <0.001	-0.007 (-0.010, -0.004)	0.241, <0.001
Yes	-0.006 (-0.010, -0.002)	0.259, 0.003	-0.007 (-0.012, -0.002)	0.147, 0.007
Current Smoker				
No			-0.006 (-0.009, -0.002)	0.211, <0.001
Yes			-0.010 (-0.015, -0.005)	0.223, <0.001

^a^ Models for females are adjusted for age, socioeconomic score, sedentary score, heart rate, anxiety score, and stress score. These models did not control for smoker status because only 2% of women were smokers.

^b^ Models for males are adjusted for age, socioeconomic score, sedentary score, smoking, heart rate, anxiety score, and stress score.

Abbreviations: DM = diabetes mellitus, HTN = hypertension, BMI = body mass index, LDL = low density lipoprotein.

Sensitivity analyses that included participants with cfPWV values at least three standard deviations from the mean and, separately, that excluded 183 participants who were genetic relatives of other participants did not materially change the results (results not shown).

## Discussion

Urbanization metrics were significantly associated with higher cfPWV. Both census data and satellite-derived land cover data suggested that the mean cfPWV was higher among participants living in urban areas compared to non-urban areas. In addition, there was an overall trend of decreasing mean cfPWV when grouping participants at 20 km intervals from the urban center. In support of these findings, linear distance from the urban center was inversely associated with mean cfPWV after controlling for standard cardiovascular risk factors, suggesting an independent association between built environment and cfPWV.

These findings are concordant with the limited body of research examining the association between urbanization and cfPWV [46–48]. One small study that considered whether urbanization is a risk factor for increased cfPWV found that individuals residing in a rural community had lower cfPWV, blood pressure, and hypertension prevalence than individuals residing in an urban community [46]. A second study reported that mean cfPWV was lower among a traditional Cameroon ethnic group following a hunter-gatherer lifestyle than among individuals of this group or Bantou farmers residing in a semi-urban area [47]. Migrants of the traditional Cameroon ethnic group to urban areas had higher cfPWV than non-migrants of this group [48]. Other than these studies, the relationship between urbanization and cfPWV has primarily been studied in relation to non-cardiovascular endpoints. For example, one study considered both a built environment index and cfPWV as risk factors for depression and coronary artery disease but did not consider the relationship between urbanization and cfPWV [49].

Our unadjusted results are also consistent with previous studies that have shown that cfPWV tends to be higher in older adults, overweight/obese individuals, and individuals with DM [15,50–52]. Male sex also seems to be a predictor of higher PWV among middle-aged, but not among older adults [53]. We controlled for these factors, and others such as smoking and LDL cholesterol levels, which were associated with both cfPWV and urbanization. Therefore, in the multivariate regression models, our measure of urbanization may serve as a proxy for unmeasured factors such as increased exposure to air pollution.

Across the study area (approximately 80 km from the western-most point to the eastern-most point), the total difference in cfPWV attributed to distance from the city center was 0.56 m/s accounting for age, SES score, sedentary score, smoking status, heart rate, anxiety score, and stress levels (Table 2). This increase in cfPWV may be physiologically meaningful. A recent meta-analysis of 17 longitudinal studies (n = 15,877) found that a 1 m/s increase in cfPWV was independently associated with a 14% increase in total cardiovascular events, a 15% increase in cardiovascular mortality, and a 15% increase in all-cause mortality [28].

The temporal relationship between cfPWV and BP is beyond the scope of this cross-sectional study; however, the functional relationship between cfPWV and BP is likely bidirectional. Previous prospective studies have found that aortic stiffness is a risk factor for increased SBP and incident HTN [22,23,54]. Conversely, arterial stiffening could be accelerated by higher SBP because of the structural and functional alternations in the walls of the central elastic arteries in response to the chronically elevated distending pressures. In our study, controlling for peripheral BP did not meaningfully change the effect estimates except among women with diabetes, high BMI, or high LDL, and among men with high LDL [55–57].

Additionally, while overall urbanization was associated with cfPWV, there was evidence in the multivariate models that age and smoker status modified the association between distance from the urban center and cfPWV as the gradient of decline in cfPWV was greater in older individuals and in smokers. Neither BMI nor new diagnosis of diabetes modified the association between urbanization and cfPWV.

Our study had several strengths. One was the use of various measures of urbanization to test the associations with cfPWV, a gold standard measure of aortic stiffness. Previous studies have considered CVD risk factors in relation to aspects of urbanization such as population size, population density, access to transportation, access to health infrastructure, economic factors, and environmental factors [3,58,59]. Less has been done to examine the relationship between urbanization defined by land cover type and CVD. By using land cover classification, we reduced exposure misclassification since we could identify smaller or developing urban enclaves. For example, we identified a rapidly urbanizing municipality approximately 65 km southwest from the urban center that had been incorrectly classified as rural based on the India Census data (Fig 1). Concordant with our hypothesis that residence in an urban area is associated with higher mean cfPWV, we found that mean cfPWV of participants in the second urban cluster had significantly higher mean cfPWV than the non-urban participants in the same distance interval.

Our study also had several limitations. While the land cover data provided benefits in terms of identifying rapidly developing urban areas, the land cover classifications did not differentiate land use within urban areas or specific components of the urban environment that may affect cfPWV. Other work is being done to classify within urban area gradients via remote sensing utilizing other built environment metrics, such as a vegetation index and impervious surfaces which could contribute to the developing evidence-base of the health impact of urban green spaces [60,61]. Another potential limitation with our analysis was the geocoding method. Geocoding participant locations can be difficult in rapidly developing areas without reliable address network systems and Global Position System (GPS) ascertainment is not viable with large sample populations. Exposure misclassification from positional error could affect our analysis at the edges of our distance interval cut points, as well as with the 500 × 500 m MODIS land cover grids. Nevertheless, in a subset of participants where we compared the geocoded location to the location recorded from a GPS, the median error was only 0.39 km which is relatively small compared to the 20 km distance categories used in our main analysis. We anticipate this error to be non-differential with respect to our outcome and therefore it would be expected to bias results towards the null.

Finally, while the PURSE-HIS includes comprehensive health data on adults aged 20 to 75 years spread over an 80 × 80 km area, the study design of our analysis was cross-sectional and we do not know how long participants had resided at their current residences. We are therefore limited in our ability to draw causal inferences for the effect of urbanization on cfPWV. PURSE-HIS is currently conducting follow-up on the study participants allowing for future longitudinal analysis. Future work may allow us to draw stronger conclusions about which aspects of the urban environment may be most important to the association with cfPWV as these analyses will specifically consider ambient and household air pollution.

## Conclusions

Participants residing in urban areas were found to have significantly higher cfPWV than those residing in non-urban areas. Furthermore, proximity to the urban center was inversely associated with mean cfPWV. The association with urbanization differed based on age, sex, and other CVD risk factors. Further studies are required to validate these findings and to determine which aspects of the built environment are most strongly associated with cfPWV.

## Supporting information

S1 File The PURSE-HIS structured instruments. (DOCX)

S2 File The PURSE-HIS data. (XLSX)
