# Supplementary material for: Urbanization as a risk factor for aortic stiffness in a cohort in India
Source: PLoS One. 2018 Aug 1;13(8):e0201036. doi: 10.1371/journal.pone.0201036 (PMC6070252; doi:10.1371/journal.pone.0201036)
Supplement: S1 File — (DOCX) [file pone.0201036.s001.docx]

**XIII. The PURSE-HIS Structured Instruments**

**1. Community Questionnaire**

- 1. **Community Type**

| Urban | Semi urban | Rural |
| --- | --- | --- |

- 1. **Community Name/Identification Label**

|  |
| --- |

- 1. **Last kilometer access**

| Path | Mud Road | Stoned Road | Tarred Road | Other |
| --- | --- | --- | --- | --- |

- 1. **Closest tarred road and distance**

| National Highway | State Highway | Main Road | Cross Road | Street | Other |
| --- | --- | --- | --- | --- | --- |

**1.5 Distance from the closest urban center/Town/City**

**1.6 Transportation Hubs present**

| **Hub** | **Frequency of Stops/day** |
| --- | --- |
| Road side bus stop |  |
| Constructed bus stop |  |
| Bus Station |  |
| Railway station |  |
| Airport |  |
| Others |  |

**1.7 Average commuting time to nearest town/urban center/city in min**

**1.8 Schools present in community**

| **School Name** | **Type** | **Strength** | **Other** |
| --- | --- | --- | --- |
|  |  |  |  |
|  |  |  |  |
|  |  |  |  |

**1.8 Health care facilities present in the community**

| **Name** | **Type** | **Beds** | **Timings** | **Others** |
| --- | --- | --- | --- | --- |
|  |  |  |  |  |
|  |  |  |  |  |
|  |  |  |  |  |
|  |  |  |  |  |
|  |  |  |  |  |

**1.9 Community Facilities Available**

| Vegetable Store |  |
| --- | --- |
| Provision Store |  |
| Bakery |  |
| Meat Store |  |
| Police Station |  |
| Fire Station |  |
| Post Office |  |
| Telephone Exchange |  |
| Water Board |  |
| Electricity Board |  |
| Petrol Bunk |  |
| LPG Outlet |  |
| Pharmacy |  |
| Park |  |
| Stadium |  |
| Swimming pool |  |
| Theater |  |
| Shopping area |  |
| Hotel |  |
| Restaurant |  |
| Temple |  |
| Other Place of Worship: | |

**1.10 Primary Water Source**

| Piped water | Bore well | Tube well | Open Well | Lake/Pond | Rain water | Others |
| --- | --- | --- | --- | --- | --- | --- |

**1.11 Electrification**

| Coverage/Penetration |  |
| --- | --- |
| Duration of Supply |  |

**1.12 Tobacco Availability in the Community**

| none | 1 store | 2 – 5 stores | 6 – 10 stores | > 10 stores |
| --- | --- | --- | --- | --- |

**1.13 Three commonest Tobacco products used in the Community**

| **Products** | **Cost/Unit** | **Quantity sold/day** |
| --- | --- | --- |
|  |  |  |
|  |  |  |
|  |  |  |

**1.14 Liquor Stores in the Community**

| none | 1 store | 2 – 5 stores | 6 – 10 stores | > 10 stores |
| --- | --- | --- | --- | --- |

**1.13 Three commonest Alcohol products used in the Community**

| **Products** | **Cost/Unit** | **Quantity sold/day** |
| --- | --- | --- |
|  |  |  |
|  |  |  |
|  |  |  |

**1.13 Three most common Fruits bought by the Community**

| **Products** | **Cost/Kg** |
| --- | --- |
|  |  |
|  |  |
|  |  |

**1.14 The Cost of the following food commodities in the community**

| **Products** | **Cost/Kg** |
| --- | --- |
| Potato |  |
| Onion |  |
| Sugar |  |
| Bread /loaf |  |
| Meat/poultry/fish |  |

**1.15 Three most food items bought by the Community**

| **Soft drinks** | **Packed Snacks** | **Frozen/ready to eat** |
| --- | --- | --- |
|  |  |  |
|  |  |  |
|  |  |  |

**PURSE-HIS**

PURSE ID

**2. Family Questionnaire**

2.1 Complete Address of the House

2.2 Total Individuals in the House

2.3 Household Individuals

| Serial No | Index | Name | Sex | DOB | Edu Level | Tobacco Use | Health Problems |
| --- | --- | --- | --- | --- | --- | --- | --- |
|  |  |  |  |  |  |  |  |
|  |  |  |  |  |  |  |  |
|  |  |  |  |  |  |  |  |
|  |  |  |  |  |  |  |  |
|  |  |  |  |  |  |  |  |
|  |  |  |  |  |  |  |  |
|  |  |  |  |  |  |  |  |
|  |  |  |  |  |  |  |  |
|  |  |  |  |  |  |  |  |

2.4 Deaths in the Family in the Previous 5 years

| Serial No | Index | Name | Sex | DOB | DOD | Edu Level | Tobacco Use | Diagnosis and Terminal event |
| --- | --- | --- | --- | --- | --- | --- | --- | --- |
|  |  |  |  |  |  |  |  |  |
|  |  |  |  |  |  |  |  |  |
|  |  |  |  |  |  |  |  |  |
|  |  |  |  |  |  |  |  |  |
|  |  |  |  |  |  |  |  |  |
|  |  |  |  |  |  |  |  |  |
|  |  |  |  |  |  |  |  |  |

2.5 Hand drawn map with directions from the nearest fixed location in the area

2.6 House Construction details. Circle appropriate

| **Roof** | **Walls** | **Flooring** |
| --- | --- | --- |
| Cement | Brick | Tiled |
| Tiles | Stone | Cement |
| Wood | Wood | Wood |
| Asbestos | Asbestos | Clay/Mud |
| Thatched | Others: | Others: |
| Others: |  |  |

**2.7 Electricity Supply**

| 19-24 Hours | 12-18 Hours | 6 – 9 hours | < 6 hours | In days | In weeks |
| --- | --- | --- | --- | --- | --- |

2.8 Primary Water Source

| Piped water | Bore well | Tube well | Open Well | Lake/Pond | Rain water | Others |
| --- | --- | --- | --- | --- | --- | --- |

2.9. Secondary Water Source

|  |
| --- |

2.9 Drinking water supply

| Unprocessed Primary water supply |
| --- |
| Primary water is boiled |
| Primary water is passed to home derived filtering process (cloth, etc) |
| Primary water is passed through candle filters |
| Primary water is passed through RO process |
| Drinking water is derived from - |

2.10 Fuel used for cooking

| Wood | Organic | LPG | Kerosene | Biogas | Electric | Solar | Others |
| --- | --- | --- | --- | --- | --- | --- | --- |

2.11 Heating source

| Coal | Wood | Organic | Gas | Electricity | Others |
| --- | --- | --- | --- | --- | --- |

2.12 Modern Appliances used for household work

| Food Processor | Grinder | Hand mixer | Electrical Hotplate | Owen | Microwave Owen |
| --- | --- | --- | --- | --- | --- |
| Refrigerator | Dish washer | Washing machine | Electrical Iron | Vacuum Cleaner |  |
| Others |  |  |  |  |  |

2.13 Mechanized Transportation used by household members

| **Type** | **Number** |
| --- | --- |
| Cycle |  |
| Moped (< 100 cc) |  |
| Motorcycle/scooter (100 cc and above) |  |
| Car |  |
| Tractor |  |

2.14 Household expenditure details

|  | **In Rs** | **Percentage** |
| --- | --- | --- |
| Total monthly household income |  |  |
| Income spent on Food |  |  |
| Income spent on Household expenses |  |  |
| Income spent on Transport |  |  |
| Income spent on Education |  |  |
| Income spent on Medical expenses |  |  |
| Others |  |  |

2.15 Household Income Source

|  | **In Rs** | **Percentage** |
| --- | --- | --- |
|  |  |  |
|  |  |  |
|  |  |  |
|  |  |  |
|  |  |  |

2.16 Family owned Agricultural Details

| Total agricultural land owned |  |
| --- | --- |
| Total Cultivable land |  |
| Type of Irrigation | Canal water |
|  | Bore well |
|  | Tube well |
|  | Open well |
|  | Rainwater |

**PURSE-HIS**

PURSE ID

**3. Subject Questionnaire**

3.1 Name

|  |
| --- |

3.2 Father’s Name

|  |
| --- |

3.3 Husband’s Name (if applicable)

|  |
| --- |

3.4 Date of Birth

|  |  |  |  |  |  |  |  |  |  |
| --- | --- | --- | --- | --- | --- | --- | --- | --- | --- |
| D | D |  | M | M |  | Y | Y | Y | Y |

3.5 Sex

| Male | Female |
| --- | --- |

3.6 Marital Status

| Unmarried | Married-Years: | Widowed | Divorced |
| --- | --- | --- | --- |

3.7 Religion and Cast

| Hindu | Cast/Division | Sub Cast/Sub Division |
| --- | --- | --- |
| Muslim |  |  |
| Christian |  |  |
| Sikh |  |  |
| Jain |  |  |
| Other: |  |  |

3.8. Highest Level of Education

| Nil |
| --- |
| Primary |
| Secondary |
| Higher Education - Professional |
| Higher Education – Arts |
| Higher Education – Others |
| Postgraduate |
| Doctoral |
| Others |
|  |

3.9 Primary Occupation with detailed description

|  |
| --- |

3.10 Secondary Occupation with detailed description

|  |
| --- |

3.11 Monthly Income Source

|  | **In Rs** | **Percentage** |
| --- | --- | --- |
| Total Monthly Income |  |  |
| Details of Income Sources | | |
|  |  |  |
|  |  |  |
|  |  |  |
|  |  |  |

3.12 Work discontinued due to

| Retirement | At Age: |
| --- | --- |
| Disability | Accident |
|  | Stroke |
|  | Aging |
|  | Chronic Infection |
|  | Polio |
|  | Leprosy |
|  | Physical Assault |
| Others |  |

3.13 Commutes to work by the following means

| **Modality** | **Duration(min)** |
| --- | --- |
|  |  |
|  |  |
|  |  |
|  |  |
|  |  |
|  |  |

**PURSE-HIS**

**4. Medical Questionnaire**

PURSE ID

**4.1 Symptoms that the subject is or has experienced in the past 1 year**

| **Symptom** |  | **Duration** | **Details** |
| --- | --- | --- | --- |
| Chest Pain |  |  |  |
| On Exertion |  |  |  |
| At Rest |  |  |  |
| Nocturnal |  |  |  |
| Breathlessness |  |  |  |
| Exertion |  |  |  |
| Lying down |  |  |  |
| Nocturnal |  |  |  |
| Wheeze |  |  |  |
| Palpitations |  |  |  |
| Exertional |  |  |  |
| Paroxysmal |  |  |  |
| Syncope/Presyncope |  |  |  |
| Fatigue/Weakness |  |  |  |
| Abdominal Distension |  |  |  |
| Leg Swelling |  |  |  |
| Reduced Urine output |  |  |  |
| Cyanosis |  |  |  |
| Fever |  |  |  |
| Cough |  |  |  |
| Expectoration |  |  |  |
| Nocturnal Cough |  |  |  |
| Cough on lying down |  |  |  |
| Embolic Phenomenon |  |  |  |
| Recurrent Respiratory Infections |  |  |  |
|  |  |  |  |
| Other Symptoms |  |  |  |
|  |  |  |  |
|  |  |  |  |
|  |  |  |  |
|  |  |  |  |
|  |  |  |  |
|  |  |  |  |

| **Symptom** | **Y/N** | **Duration** | **Details** |
| --- | --- | --- | --- |
| Sudden loss of vision in one eye |  |  |  |
| Sudden onset of weakness of one half of the body |  |  |  |
| Sudden loss of sensation or numbness of one of half of the body |  |  |  |
| Sudden loss of speech |  |  |  |
| Sudden onset of difficulty in understanding the words |  |  |  |
| Sudden onset of difficulty in identifying objects or naming the objects |  |  |  |
| Sudden onset of difficulty in recognizing familiar faces |  |  |  |
| Sudden onset of difficulty in reading or writing |  |  |  |
| Sudden onset of difficulty in walking (Swaying) |  |  |  |
| Sudden onset of deviation of angle of mouth to any side |  |  |  |
| Sudden onset of vertigo with tremulousness of hands, dysphagia and dysarthria |  |  |  |
| Sudden loss of consciousness |  |  |  |
| Sudden onset of uni lateral headache, vomiting, flashes of light with blurring of vision |  |  |  |
| Family history of stroke |  |  |  |
|  |  |  |  |
|  |  |  |  |
|  |  |  |  |

**4.2 Claudicating Pain**

| **Location** | **Distance** | **Duration** |
| --- | --- | --- |
| Calf |  |  |
| Thigh |  |  |
| Buttocks |  |  |
| Other Sites |  |  |
|  |  |  |
|  |  |  |
|  |  |  |

**4.3 Symptoms suggestive of Critical Limb Ischemia**

| **Symptom** | **Duration** | **Description** |
| --- | --- | --- |
| Rest Pain |  |  |
| Non Healing Ulcers |  |  |
| Gangrene |  |  |
| Amputation |  |  |

**4.4 Hypertension History**

| First Detected in (year) |  | Details |
| --- | --- | --- |
| Presenting complaint |  |  |
| Place of Detection |  |  |
| Evidence of Target Organ Damage | LVH |  |
|  | Retinopathy |  |
|  | Nephropathy |  |
|  | Complications |  |
| Medication used |  |  |
| Compliance |  |  |
| Family History |  |  |

**4.5 Diabetic History**

| First Detected in (year) |  | Details |
| --- | --- | --- |
| Presenting complaint |  |  |
| Place of Detection |  |  |
| Evidence of Target Organ Damage | Retinopathy |  |
|  | Nephropathy |  |
|  | Neuropathy |  |
|  | Gastropathy |  |
|  | Autonomic |  |
| Medication used |  |  |
| Compliance |  |  |
| Family History |  |  |

**4.6 Past Medical History/ Relevant Family History**

|  |
| --- |

**4.7 Admissions in the past**

| **Diagnosis** | **Admitted at** | **DOA** | **DOD** | **Cost** |
| --- | --- | --- | --- | --- |
|  |  |  |  |  |
|  |  |  |  |  |
|  |  |  |  |  |
|  |  |  |  |  |

**4.8 Medications being used at present**

| **Form** | **Name** | **Dose** | **Route** | **Frequency** |
| --- | --- | --- | --- | --- |
|  |  |  |  |  |
|  |  |  |  |  |
|  |  |  |  |  |
|  |  |  |  |  |
|  |  |  |  |  |
|  |  |  |  |  |
|  |  |  |  |  |
|  |  |  |  |  |
|  |  |  |  |  |
|  |  |  |  |  |
|  |  |  |  |  |
|  |  |  |  |  |

**4.9 Smoking/Tobacco Consumption History**

| Active Smoker | Ex Smoker | Passive Smoker |
| --- | --- | --- |
| Duration | From: | To: |
| Type of Tobacco used |  |  |
| Per day consumption |  |  |

**4.10 Alcohol Consumption History**

| Duration | From: | To: |
| --- | --- | --- |
| Type of Alcohol |  |  |
| Per day consumption |  |  |

**4.11 Other Addictions**

| Pann |
| --- |
| Supari |
| Gutka |
|  |
|  |

**4.12 Appetite**

| Normal | Increased | Decreased | Reason |
| --- | --- | --- | --- |

**4.13 Sleep**

| Normal | Increased | Decreased | Reason |
| --- | --- | --- | --- |
| Sleeps at |  | Awakes at |  |

**4.14 Micturation**

| **Times in Day** | **Times after Sleeping** | **Problems** |
| --- | --- | --- |
|  |  |  |

**4.15 Defecation**

| **Frequency** | **Color** | **Consistency** | **Others** |
| --- | --- | --- | --- |
|  |  |  |  |

**4.16 High Risk Behavior**

|  |
| --- |

**4.17** **Menstrual History**

| Age of Menarche |  |
| --- | --- |
| Age of Menopause |  |
| Duration of Cycle |  |
| Duration of Bleeding |  |
| Quantity |  |
| Other |  |

**4.18 Contraception Usage**

| **Type** | **From** | **To** | **Compliants** |
| --- | --- | --- | --- |
|  |  |  |  |
|  |  |  |  |
|  |  |  |  |

**4.19 Obstetric History**

| **No** | **Gestation** | **Delivery** | **Sex** | **Breast Fed** | **Complications** |
| --- | --- | --- | --- | --- | --- |
|  |  |  |  |  |  |
|  |  |  |  |  |  |
|  |  |  |  |  |  |
|  |  |  |  |  |  |
|  |  |  |  |  |  |
|  |  |  |  |  |  |

**4.20 Other Relevant History**

|  |
| --- |

**4.21** **General Physical Examination**

| Decubitus |  |
| --- | --- |
| Orthopnoea |  |
| Tacypnoea |  |
| Clubbing |  |
| Central Cyanosis |  |
| Peripheral Cyanosis |  |
| Pallor |  |
| Icterus |  |
| Edema |  |
| Any Other Abnormalities |  |

**4.22 Radial Pulse Examination**

| Rate |  |
| --- | --- |
| Rhythm |  |
| Volume |  |
| Character |  |
| Radio Radial Delay |  |
| Radio Femoral Delay |  |
| Condition of Vessel Wall |  |
| Other |  |

**4.23 Peripheral Pulse Examination**

| **Location** | **Right** | **Left** |
| --- | --- | --- |
| Carotid |  |  |
| Temporal |  |  |
| Brachial |  |  |
| Radial |  |  |
| Ulnar |  |  |
| Femoral |  |  |
| Popletial |  |  |
| Posterior Tibial |  |  |
| Dorsalis Pedis |  |  |

**4.24 Blood Pressure Measurement**

| **Location** |  | **Right** | **Left** |
| --- | --- | --- | --- |
| Upper Limb | Systolic |  |  |
|  | Diastolic |  |  |
|  |  |  |  |
| Lower Limb | Systolic |  |  |
|  | Diastolic |  |  |

**4.25** **JVP Measurement**

| Mean Height in cm |  |
| --- | --- |
| a Wave |  |
| c Wave |  |

**4.26 Lower Limb Examination**

| Dependent Rubor |  |
| --- | --- |
| Pallor On Elevation |  |
| Absence Of Hair Growth |  |
| Dystrophic Toenails |  |
| Cool, Dry, Fissured Skin |  |
| Interdigital Spaces |  |
| Fissures |  |
| Ulcerations |  |
| Infections |  |
| Other | |

**4.27 Cardiac Examination**

| **Inspection** | Apical Impulse |  |
| --- | --- | --- |
|  | Precordial bulge |  |
|  | P A Pulsation |  |
|  | Parasternal lift |  |
|  | Any Other |  |
|  | | |
| **Palpation** | Apex Beat |  |
|  | P.A. Pulsation |  |
|  | Palpable P2 |  |
|  | Parasternal lift |  |
|  | Thrill |  |
|  | | |
| **Percussion** | Upper Sternum |  |
|  | Lower Sternum |  |
|  | 2^nd^ Left ICS |  |
|  | Left Border |  |
|  | Right Border |  |
|  | | |
| **Auscultation** | S1 |  |
|  | S2 |  |
|  | P2 |  |
|  | Added Sounds |  |
|  | Murmurs |  |
|  |  |  |
|  |  |  |
|  |  |  |
|  |  |  |
|  |  |  |

**4.28 Neurological Examination**

| **Mental Status Examination** | Handedness – Right or left |  |
| --- | --- | --- |
|  | Level of consciousness – |  |
|  | Memory – |  |
|  | Attention |  |
|  | Judgment |  |
|  | Calculation |  |
|  | Language – word output |  |
|  | Fluency |  |
|  | Comprehension |  |
|  | Repetition |  |
|  | Reading |  |
|  | Writing |  |
|  | Naming |  |
|  | | |
| **Cranial Nerve Examination** | 1. |  |
|  | 2. |  |
|  | 3. |  |
|  | 4. |  |
|  | 6. |  |
|  | 5. |  |
|  | 7. |  |
|  | 8. |  |
|  | 9. |  |
|  | 10. |  |
|  | 11. |  |
|  | 12. |  |

|  | | |
| --- | --- | --- |
| **Motor System Exam** | Nutrition |  |
|  | Tone |  |
|  | Power |  |
|  | Reflexes |  |
|  | Co-ordination |  |
|  | Involuntary movements |  |
|  | Feel of the muscle |  |
|  | Gait |  |
|  | | |
| **Sensory System Examination** | Pain |  |
|  | Temperature |  |
|  | Touch |  |
|  | Vibration |  |
|  | Joint sense |  |
|  | Position sense |  |
|  | Romberg’s sign |  |
|  | Tactile localization |  |
|  | Tactile Discrimination |  |
|  | Stereognosis |  |
|  | Figure writing |  |
|  |  |  |
| **Cerebellar Examination** | Nystagmus |  |
|  | Intention Tremor |  |
|  | Rebound phenomenon |  |
|  | Dysarthria |  |
|  | Past pointing |  |
|  | Tandem gait |  |
|  |  |  |
| Meningeal Signs | | |
| Cranium & Spine | | |

**4.29 Fundus Examination**

|  |
| --- |

**PURSE-HIS**

**5. Anthropometric Questionnaire**

PURSE ID

| **Variable** | **Measurement** |
| --- | --- |
| Waist circumference |  |
| Weight |  |
| Hip circumference |  |
| Height |  |
| Mid upper right arm circumference |  |
| Right calf circumference |  |
| Head circumference |  |
| Upper flexed arm circumference |  |
| Right arm triceps skin fold |  |
| Right calf skin fold |  |
| Biceps skinfold |  |
| Subscapular skinfold |  |
| Supra spinal skinfolds |  |
| Breadth of humerus |  |
| Breadth of femur |  |

**PURSE-HIS**

**6. Stress Questionnaire**

PURSE ID

| 1 | I can take a measured look at a job to be done with feeling an urge to rush into action before getting the thing properly through out | **YES** | **NO** |
| --- | --- | --- | --- |
| 2 | I have recently had to give up an important personal relationship | **YES** | **NO** |
| 3 | I often need understanding friends to cheer me up | **YES** | **NO** |
| 4 | I can control my temper; when I lose it, this is calculated and I don't go beyond what I intend to say or do | **YES** | **NO** |
| 5 | I find it easy to get along with people who hold different points of view to my own | **YES** | **NO** |
| 6 | My mood often goes up and down | **YES** | **NO** |
| 7 | Having to tolerate delays of any kind is very irritating to me | **YES** | **NO** |
| 8 | I prefer to assume complete responsibility rather than share it with others | **YES** | **NO** |
| 9 | Recent events in my life have forced an important change in my social relationship | **YES** | **NO** |
| 10 | I do not suffer fool gladly | **YES** | **NO** |
| 11 | I can focus on one thing when necessary and clear my mind of other thing to be done | **YES** | **NO** |
| 12 | I take pride in getting the job done faster than most | **YES** | **NO** |
| 13 | I sometimes feel “just miserable” for no good reasons | **YES** | **NO** |
| 14 | My feelings rather easily hurt | **YES** | **NO** |
| 15 | Deadlines is very important to me | **YES** | **NO** |
| 16 | I can say “no” to people who make an unreasonable demand without making them upset | **YES** | **NO** |
| 17 | My sexual needs are largely satisfied | **YES** | **NO** |
| 18 | I usually try to deal with problems systematically and in an organized way | **YES** | **NO** |
| 19 | I have plenty of battles on my hands at work | **YES** | **NO** |
| 20 | I have suffered considerably from constant argument at home or at work | **YES** | **NO** |
| 21 | I have very real financial problems | **YES** | **NO** |
| 22 | I am often troubled by feelings of guilt | **YES** | **NO** |
| 23 | I can get over disappointments without getting out upset; I realize that one cannot have everything the way one wishes | **YES** | **NO** |
| 24 | I would call myself tense or “highly strung” | **YES** | **NO** |
| 25 | I can immerse myself in constructive activity as a way of talking my mind away from problems in a close relationship | **YES** | **NO** |
| 26 | I have recently had serious problems in a close relationship | **YES** | **NO** |
| 27 | I can usually get other people to see all sides of problems | **YES** | **NO** |
| 28 | I can unwind quickly on a holiday and begin to enjoy myself from the start | **YES** | **NO** |
| 29 | I enjoy competing at work and else where | **YES** | **NO** |
| 30 | I feel I am as good as the next person | **YES** | **NO** |
| 31 | When confronted with a problem I usual remain optimistic about the outcome | **YES** | **NO** |
| 32 | I get impatient and angry about incompetence and inefficiency | **YES** | **NO** |
| 33 | I get attack of shaking of trembling | **YES** | **NO** |
| 34 | I work long hours from choice | **YES** | **NO** |
| 35 | I or members of my family have recently experienced problems due to illness | **YES** | **NO** |
| 36 | I am irritable person | **YES** | **NO** |
| 37 | I drive myself harder than most | **YES** | **NO** |
| 38 | I can usually breakdown a problem into manageable chunks | **YES** | **NO** |
| 39 | I worry about awful things that might happen | **YES** | **NO** |
| 40 | I would call myself as a nervous person | **YES** | **NO** |
| 41 | I can count on the support of my family and friends | **YES** | **NO** |
| 42 | I have to spend too much time away from home | **YES** | **NO** |
| 43 | I am very ambitious | **YES** | **NO** |
| 44 | I am easily hurt when people find fault with me or with my work | **YES** | **NO** |
| 45 | I some time have to assume responsibility for events over which I have no control | **YES** | **NO** |
| 46 | I am troubled by feeling of inferiority | **YES** | **NO** |
| 47 | Some one close to me has recently died | **YES** | **NO** |
| 48 | At times I have more work than I have able to cope with | **YES** | **NO** |
| 49 | I am able to tell other people what I feel and think; I do not simmer privately explode | **YES** | **NO** |
| 50 | I tend to get involved in my different ideas and project | **YES** | **NO** |
| 51 | I suffer from sleeplessness | **YES** | **NO** |
| 52 | I have to work with others of unpredictable and uncertain temperament | **YES** | **NO** |
| 53 | I feel dissatisfied at work due to, e.g. blocked promotion, threat of redundancy, excessive demands of superiors, etc. | **YES** | **NO** |

**PURSE-HIS**

**7. Anxiety Questionnaire**

PURSE ID

**1. Anxious mood** This item covers the emotional condition of uncertainty about the future, ranging from worry, insecurity, irritability and apprehension to overpowering dread.

| **0** | The patient is neither more or less insecure or irritable than usual. |
| --- | --- |
| **1** | Doubtful whether the patient is more insecure or irritable than usual. |
| **2** | The patient expresses more clearly to be in a state of anxiety, apprehension or irritability, which he may find difficult to control. However, the worrying still is about minor matters and thus without influence on the patient's daily life. |
| **3** | At times the anxiety or insecurity is more difficult to control because the worrying is about major injuries or harms which might occur in the future. Has occasionally interfered with the patient's daily life. |
| **4** | The feeling of dread is present so often that it markedly interferes with the patient's daily life. |

**2. Tension** This item includes inability to relax, nervousness, bodily tensions, trembling and restless fatigue.

| **0** | The patient is neither more nor less tense than usual |
| --- | --- |
| **1** | The patient seems somewhat more nervous and tense than usual. |
| **2** | Patient expresses clearly unable to relax and full of inner unrest, which he finds difficult to control, but it is still without influence on the patient's daily life. |
| **3** | The inner unrest and nervousness is so intense or frequent that it occasionally interferes with the patient's daily work. |
| **4** | Tensions and unrest interfere with the patient's life and work at all times. |

**3. Fears** This item includes fear of being in a crowd, of animals, of being in public places, of being alone, of traffic, of strangers, of dark etc. It is important to note whether there has been more phobic anxiety during the present episode than usual.

| **0** | Not present. |
| --- | --- |
| **1** | Doubtful whether present. |
| **2** | The patient experiences phobic anxiety but is able to fight it. |
| **3** | It is difficult to fight or overcome the phobic anxiety, which thus to some extent interferes with the patient's daily life and work. |
| **4** | The phobic anxiety clearly interferes with the patient's daily life and work. |

**4.Insomnia** This item covers the patient's subjective experience of sleep duration and sleep depth during the three preceding nights. Note: Administration of hypnotics or sedatives is disregarded

| **0** | Usual sleep duration and sleep depth |
| --- | --- |
| **1** | Sleep duration is doubtfully or slightly reduced (e.g. due to difficulties falling asleep), but no change in sleep depth. |
| **2** | Sleep depth is also reduced, sleep being more superficial. Sleep as a whole is somewhat disturbed. |
| **3** | Sleep duration and sleep depth is markedly changed. Sleep periods total only a few hours per 24 hours. |
| **4** | Sleep depth is so shallow that the patient speaks of short periods of slumber or dozing, but no real sleep. |

**5.Difficulties in concentration and memory** This item covers difficulties in concentration, making decision about everyday matters, and memory

| **0** | The patient has neither more nor less difficulty in concentration and/or memory that usual. |
| --- | --- |
| **1** | Doubtful whether the patient has difficulty in concentration and/or memory. |
| **2** | Even with a major effort it is difficult for the patient to concentrate on his daily routine work. |
| **3** | The patient has pronounced difficulties with concentration, memory, or decision making, e.g. in reading a newspaper article or watching a television programme to the end. |
| **4** | During the interview the patient shows difficulty in concentration, memory or decision making. |

**6.Depressed mood** This item covers both the verbal and the non-verbal communication of sadness, depression, despondency, helplessness and hopelessness

| **0** | Not present. |
| --- | --- |
| **1** | Doubtful whether the patient is more despondent or sad than usual, or is only vaguely so. |
| **2** | The patient is more clearly concerned with unpleasant experiences, although he still lacks helplessness or hopelessness. |
| **3** | The patient shows clear non-verbal signs of depression and/or hopelessness. |
| **4** | The patient remarks on despondency and helplessness or the non-verbal signs dominate the interview and the patient cannot be distracted. |

**7. Generalsomatic symptoms**: **Muscular** Weakness, stiffness, soreness or real pain, more or less diffusely localized in the muscles, such as jaw ache or neck ache.

| **0** | The patient is neither more nor less sore or stiff in the muscles than usual. |
| --- | --- |
| **1** | The patient seems somewhat more stiff or sore in the muscles than usual. |
| **2** | The symptoms have the character of pain. |
| **3** | Muscle pain interferes to some extent with the patient's daily work and life. |
| **4** | Muscle pain is present most of the time and clearly interferes with the patient's daily work and life |

**8. General somatic symptoms: Sensory** This item includes increased fatigability and weakness or real functional disturbances of the senses, including tinnitus, blurring of vision, hot and cold flashes and prickling sensations

| **0** | Not present. |
| --- | --- |
| **1** | Doubtful whether the patient's indications of symptoms are more pronounced than usual |
| **2** | The sensations of pressure reach the character of buzzing in the ears, visual disturbances and prickling or itching sensations in the skin. |
| **3** | The generalized sensory symptoms interfere to some extent with the patient's daily life and work. |
| **4** | The generalized sensory symptoms are present most of the time and clearly interfere with the patient's daily life and work. |

**9.Cardiovascular symptoms** This item includes tachycardia, palpitations, oppression, chest pain, throbbing in the blood vessels, and feelings of faintness.

| **0** | Not present. |
| --- | --- |
| **1** | Doubtful whether present. |
| **2** | Cardiovascular symptoms are present, but the patient can still control them. |
| **3** | The patient has occasional difficulty controlling the cardiovascular symptoms, which thus to some extent interfere with his daily life and work. |
| **4** | Cardiovascular symptoms are present most of the time and clearly interfere with the patient's daily life and work. |

**10.Respiratory symptoms** Feelings of constriction or contraction in throat or chest, dyspnoea or choking sensations and sighing respiration

| **0** | Not present. |
| --- | --- |
| **1** | Doubtful whether present. |
| **2** | Respiratory symptoms are present, but the patient can still control them. |
| **3** | The patient has occasional difficulty controlling the respiratory symptoms, which thus to some extent interfere with his daily life and work. |
| **4** | Respiratory symptoms are present most of the time and clearly interfere with the patient's daily life and work. |

**11. Gastro-intestinal symptoms** This item covers difficulties in swallowing, "sinking" sensation in stomach, dyspepsia (heartburn or burning sensation in the stomach, abdominal pains related to meals, fullness, nausea and vomiting), abdominal rumbling and diarrhoea.

| **0** | Not present. |
| --- | --- |
| **1** | Doubtful whether present (or doubtful whether different from usual). |
| **2** | One or more gastro-intestinal symptoms are present, but the patient can still control them. |
| **3** | The patient has occasional difficulty controlling the gastro-intestinal symptoms, which to some extent interfere with his daily life and work. |
| **4** | The gastro-intestinal symptoms are present most of the time and interfere clearly with the patient's daily life and work |

**12.Genito-urinary symptoms** This item includes non-organic or psychic symptoms such as frequent or more pressing passing of urine, menstrual irregularities, anorgasmia, dyspareunia, premature ejaculation, loss of erection.

| **0** | Not present. |
| --- | --- |
| **1** | Doubtful whether present (or doubtful whether different from usual) |
| **2** | One or more genito-urinary symptoms are present, but do not interfere with the patient's daily life and work. |
| **3** | Occasionally, one or more genito-urinary symptoms are present to such a degree that they interfere to some extent with the patient's daily life and work. |
| **4** | The genito-urinary symptoms are present most of the time and interfere clearly with the patient's daily life and work. |

**13. Other autonomic symptoms** This item includes dryness of the mouth, blushing or pallor, sweating and dizziness

| **0** | Not present. |
| --- | --- |
| **1** | Doubtful whether present. |
| **2** | One or more autonomic symptoms are present, but they do not interfere with the patient's daily life and work. |
| **3** | Occasionally, one or more autonomic symptoms are present to such a degree that they interfere to some extent with the patient's daily life and work. |
| **4** | Autonomic symptoms are present most of the time and clearly interfere with the patient's daily life and work. |

**14.Behaviour during interview** The patient may appear tense, nervous, agitated, restless, tremulous, pale, hyperventilating or sweating during the interview. Based on such observations a global estimate is made.

| **0** | The patient does not appear anxious. |
| --- | --- |
| **1** | It is doubtful whether the patient is anxious. |
| **2** | The patient is moderately anxious. |
| **3** | The patient is markedly anxious. |
| **4** | Patient is overwhelmed by anxiety, for example with shaking and trembling all over. |

**PURSE-HIS**

**8. Depression Questionnaire**

PURSE ID

**1. Depressed Mood** (sadness, hopelessness,helplessness, worthlessness)

| **0** | Absent |
| --- | --- |
| **1** | These feeling states indicated only on questioning |
| **2** | These feeling states reported verbally |
| **3** | Communicates feeling states nonverbally |
| **4** | Reports only these feeling states in spontaneous verbal and nonverbal communication |

**2. Feelings of Guilt**

| **0** | Absent |
| --- | --- |
| **1** | Self-reproach, feels he/she has let people down |
| **2** | Ideas of guilt or rumination over past errors or “sinful” deeds |
| **3** | Present illness is a punishment; delusions of guilt |
| **4** | Hears accusatory or denunciatory voices and/or experiences threatening visual hallucinations |

**3. Suicide**

| **0** | Absent |
| --- | --- |
| **1** | Feels life is not worth living |
| **2** | Wishes he/she were dead or has any thoughts of possible death to self |
| **3** | Suicidal ideas or gestures |
| **4** | Attempts at suicide (any serious attempt rates “4”) |

**4. Insomnia – Early**

| **0** | No difficulty falling asleep |
| --- | --- |
| **1** | Complains of occasional difficulty falling asleep (i.e., >1/2 hour) |
| **2** | Complains of nightly difficulty falling asleep |

**5. Insomnia – Middle**

| **0** | Absent |
| --- | --- |
| **1** | No difficulty |
| **2** | Complains of being restless and disturbed during the night |
| **3** | Wakes during the night – getting out of bed rates “2” (except for purposes of voiding |

**6. Insomnia – Late**

| **0** | No difficulty |
| --- | --- |
| **1** | Wakes in early hours of morning but falls back asleep |
| **2** | Unable to fall asleep again if he/she gets out of bed |

**7. Work and Activities**

| **0** | No difficulty |
| --- | --- |
| **1** | Thoughts of incapacity; fatigue or weakness related to activities, work or hobbies |
| **2** | Loss of interest in activity, hobbies or work – either directly reported by patient or indirectly in listlessness, indecision and vacillation (feels he/she has to push self to work or for activities) |
| **3** | Decrease in actual time spent in activities or decrease in productivity |
| **4** | Stopped working because of present illness |

**8. Retardation** (slowness of thought and speech; impaired ability to concentrate; decreased motor activity)

| **0** | Normal speech and thought |
| --- | --- |
| **1** | Slight retardation at interview |
| **2** | Oblivious retardation at interview |
| **3** | Interview difficult |
| **4** | Complete stupor |

**9. Agitation**

| **0** | None |
| --- | --- |
| **1** | Fidgetiness |
| **2** | “Playing with” hands, hair, etc |
| **3** | Moving about, can’t sit still |
| **4** | Hand wringing, nail biting, hair pulling, lip biting |

**10. Anxiety – Psychic**

| **0** | No difficulty |
| --- | --- |
| **1** | Subjective tension and irritability |
| **2** | Worries about minor matters |
| **3** | Apprehensive attitude apparent in face or speech |
| **4** | Fears expressed without questioning |

**11. Anxiety – Somatic**

| **0** | Absent |
| --- | --- |
| **1** | Mild |
| **2** | Moderate |
| **3** | Severe |
| **4** | Incapacitating |

**12. Somatic Symptoms** – Gastrointestinal

| **0** | None |
| --- | --- |
| **1** | Loss of appetite, but eating; heavy feeling in abdomen |
| **2** | Difficulty eating without urging; requests or requires laxatives or medication for bowels or medication for GI symptoms |

**13. Somatic Symptoms** – General

| **0** | None |
| --- | --- |
| **1** | Heaviness in limbs, back of head; backache, headache, muscle ache; loss of energy  and fatigue |
| **2** | Any clear-cut symptoms rate“2” |

**14. Genital Symptoms** (i.e., loss of libido, menstrual disturbances)

| **0** | Absent |
| --- | --- |
| **1** | Mild |
| **2** | Severe |

**15. Hypochondriasis**

| **0** | Not present |
| --- | --- |
| **1** | Self-absorption (bodily) |
| **2** | Preoccupation with health |
| **3** | Frequent complaints, requests for help, etc |
| **4** | Hypochondriacal delusions |

**16. Weight Loss**

| **0** | No weight loss |
| --- | --- |
| **1** | Slight or doubtful weight loss |
| **2** | Obvious or severe weight loss |

**17. Insight**

| **0** | Acknowledges being depressed or ill |
| --- | --- |
| **1** | Acknowledges illness but attributes cause to bad food, climate, overwork, virus, need for rest, etc. |
| **2** | Denies being ill at all |

**18. Diurnal Variation**

| **0** | No variation |
| --- | --- |
| **1** | Mild: doubtful or slight variation |
| **2** | Severe: clear or marked variation; if applicable, note whether symptoms are worse in  AM( ) or PM( ) |

**19. Depersonalization and Derealization** (feelings of unreality, nihilistic ideas)

| **0** | Absent |
| --- | --- |
| **1** | Mild |
| **2** | Moderate |
| **3** | Severe |
| **4** | Incapacitating |

**20. Paranoid Symptoms**

| **0** | None |
| --- | --- |
| **1** | Suspicious |
| **2** | Ideas of reference |
| **3** | Delusions of reference or persecution |
| **4** | Paranoid hallucinations |

**21. Obsessive/Compulsive Symptoms**

| **0** | Absent |
| --- | --- |
| **1** | Mild |
| **2** | Severe |

**PURSE-HIS**

**9.Physical Activity Questionnaire**

PURSE ID

| **Physical Activity** | | | |
| --- | --- | --- | --- |
| Next I am going to ask you about the time you spend doing different types of physical activity in a typical week. Please answer these questions even if you do not consider yourself to be a physically active person.  Think first about the time you spend doing work. Think of work as the things that you have to do such as paid or unpaid work, study/training, household chores, harvesting food/crops, fishing or hunting for food, seeking employment.*.* In answering the following questions 'vigorous-intensity activities' are activities that require hard physical effort and cause large increases in breathing or heart rate, 'moderate-intensity activities' are activities that require moderate physical effort and cause small increases in breathing or heart rate. | | | |
| **Questions** | | | **Response** |
|  | | | |
| 1 | Does your work involve vigorous-intensity activity that causes large increases in breathing or heart rate like *[carrying or lifting heavy loads, digging or construction work*] for at least 10 minutes continuously? | | Yes  No |
| 2 | In a typical week, on how many days do you do vigorous intensity activities as part of your work? | | Number of days |
| 3 | How much time do you spend doing vigorous-intensity activities at work on a typical day? | | Hours : minutes  Hrs Mins |
| 4 | Does your work involve moderate-intensity activity that causes small increases in breathing or heart rate such as brisk walking *[or carrying light loads*] for at least 10 minutes continuously? | | Yes  No |
| 5 | In a typical week, on how many days do you do moderate intensity activities as part of your work? | | Number of days |
| 6 | How much time do you spend doing moderate-intensity activities at work on a typical day? | | Hours : minutes  Hrs Mins |
| **Travel to and from places** | | | |
| The next questions exclude the physical activities at work that you have already mentioned. Now I would like to ask you about the usual way you travel to and from places. For example to work, for shopping, to market, to place of worship. [insert other examples if needed] | | | |
| 7 | | Do you walk or use a bicycle (*pedal cycle*) for at least 10 minutes continuously to get to and from places? | Yes  No |
| 8 | | In a typical week, on how many days do you walk or bicycle for at least 10 minutes continuously to get to and from places? | Number of days |
| 9 | | How much time do you spend walking or bicycling for travel on a typical day? | Hours : minutes  Hrs Mins |
| **Recreational Activities** | | | |
| The next questions exclude the work and transport activities that you have already mentioned.  Now I would like to ask you about sports, fitness and recreational activities (leisure), [insert relevant terms]. | | | |
| 10 | | Do you do any vigorous-intensity sports, fitness or recreational (*leisure*) activities that cause large increases in breathing or heart rate like [*running or football,]* for at least 10 minutes  Continuously? | Yes  No |
| 11 | | In a typical week, on how many days do you do vigorous intensity sports, fitness or recreational (*leisure*) activities? | Number of days |
| 12 | | How much time do you spend doing vigorous-intensity sports, fitness or recreational activities on a typical day? | Hours : minutes  Hrs Mins |

| **Physical Activity (recreational activities)** | | |
| --- | --- | --- |
| **Questions** | | **Response** |
| 13 | Do you do any moderate-intensity sports, fitness or recreational *(leisure*) activities that causes a small increase in breathing or heart rate such as brisk walking*,*(*cycling, swimming, volleyball*)for at least 10 minutes continuously | Yes 1  No 2 |
| 14 | n a typical week, on how many days do you do moderate-intensity sports, fitness or recreational (*leisure*) activities? | Number of days |
| 15 | How much time do you spend doing moderate-intensity sports, fitness or recreational (*leisure*) activities on a typical day? | Hours : minutes  Hrs Mins |
| **Sedentary Behavior** | | |
| The following question is about sitting or reclining at work, at home, getting to and from places, or with friends including time spent [sitting at a desk, sitting with friends, traveling in car, bus, train, reading, playing cards or watching television], but do not include time spent sleeping. | | |
| 16 | How much time do you usually spend sitting or reclining  on a typical day? | Hours : minutes  Hrs Mins |

**PURSE-HIS**

**10. Nutrition Questionnaire**

PURSE ID

**24 - HOUR RECALL**

| **Time** | **Food items** | **Qty** | **Energy**  **(gm)** | **Protein**  **(gm)** | **CHO**  **(gm)** | **Fats**  **(gm)** | **Sodium**  **(mg)** | **Fiber**  **(gm)** |
| --- | --- | --- | --- | --- | --- | --- | --- | --- |
|  |  |  |  |  |  |  |  |  |
| TOTAL |  |  |  |  |  |  |  |  |
| Excess  **Deficit** |  |  |  |  |  |  |  |  |

**FOOD FREQUENCY**

| **FOOD GROUPS** | **EXCESS** | **ADEQUATE** | **DEFICIT** |
| --- | --- | --- | --- |
| Cereal |  |  |  |
| Pulses/Legumes |  |  |  |
| Vegetable  i)Green leafy vegetbles  ii)Roots and Tubers  iii) Other vegetables |  |  |  |
| Fruits |  |  |  |
| Milk and milk products |  |  |  |
| Meat and poultry |  |  |  |
| Fats and oils |  |  |  |
| Sugar and jaggery |  |  |  |
